# Subjective Cognitive Decline in Brazilian Adults: Prevalence and Associated Social, Lifestyle, and Health-Related Factors: A Nationally Representative Cross-Sectional Analysis from the ELSI-Brazil Cohort

**DOI:** 10.3390/neurolint18030042

**Published:** 2026-02-24

**Authors:** Johnnatas Mikael Lopes, Paola Bertuccio, Lorenzo Blandi, Riccardo Vecchio, Anna Odone

**Affiliations:** 1 Department of Public Health, Experimental and Forensic Medicine, University of Pavia, 27100 Pavia, Italy; johnnatas.lopes@univasf.edu.br (J.M.L.); paola.bertuccio@unipv.it (P.B.); riccardo.vecchio03@universitadipavia.it (R.V.); 2 Department of Medicine, Federal University of the São Francisco Valley, Paulo Afonso 48200-000, BA, Brazil; 3 School of Public Health, Vita-Salute San Raffaele University, 20132 Milan, Italy; blandi.lorenzo@hsr.it; 4 Medical Direction, IRCCS Istituti Clinici Scientifici Maugeri, 27100 Pavia, Italy; 5 Medical Direction, Fondazione IRCCS Policlinico San Matteo, 27100 Pavia, Italy

**Keywords:** cognitive decline, dementia, risk factors, middle income country, Brazil, observational study, population-based study

## Abstract

**Background/Objectives**: Subjective cognitive decline (SCD) is an early stage of dementia, although its risk factors remain unclear. We estimated the prevalence of SCD and its associated dementia risk factors in Brazilian adults. **Methods**: This cross-sectional study is based on data from the second wave (2019–2021) of the Brazilian longitudinal study of aging (ELSI-Brazil) and a nationally representative sample of adults aged ≥50 years. Prevalence of SCD was estimated and defined as self-reported cognitive decline without objective impairment or dementia diagnosis, and the adjusted odds ratios (OR) with 95% confidence intervals (CI) were estimated through logistic regression models. **Results**: Of 6631 participants, 57.5% were women, and 54.4% were non-white, with a mean age of 65.1 years (standard deviation: ±9.70). SCD prevalence was 19.7% (95% CI 18.6–20.9) for a total of 1346 individuals. Significantly strong positive associations with SCD were observed for sociodemographic factors, particularly lower education (OR = 2.79, 95% CI: 2.02–3.85), as well as older age, non-white ethnicity, and lower income (ORs ranging from 1.50 to 1.79). Lifestyle factors, including loneliness and sedentary behavior, showed moderate associations (OR = 1.33 and 1.35, respectively). Among health-related conditions, multimorbidity was significantly associated with higher odds of SCD (OR = 1.40 for ≥3 chronic diseases), with the strongest association observed for hearing loss (OR = 2.29, 95% CI: 1.93–2.71). Diabetes, visual loss, and depressive symptoms showed more modest significant associations (OR 1.25 to 1.31). **Conclusions**: Our findings support the prioritization of vulnerable populations in public health strategies aimed at promoting healthy ageing and reducing social and health inequalities. Longitudinal studies are needed to clarify whether modifying associated factors may influence SCD trajectories.

## 1. Introduction

Cognitive decline represents one of the most relevant health concerns in aging populations worldwide [1], and it ranges from self-perceived conditions, such as the subjective cognitive decline (SCD) [2], to an objective clinical change, including mild cognitive impairment (MCI) or dementia [3].

SCD is defined as a self-reported state of worsening cognitive capacity with normal age-, gender-, and education-adjusted performance on standardized cognitive tests [4]. This condition is confirmed in the absence of other psychiatric disorders, such as major depression, which can influence the cognitive domains [4]. SCD is considered an early stage of cognitive impairment [3], offering a time-window for prevention. A recent paper from the Framingham Heart Study estimated that individuals with SCD have a 57% higher risk of developing MCI over an average period of 4.6 years, and a nearly threefold higher risk of dementia over 6.8 years, even after adjusting for genetic factors [5]. Those who progress to cognitive disorders seem to have a new onset of SCD within 20 years, which becomes more noticeable in the last four years before diagnosis. Those who do not progress to cognitive disorders report SCD since younger age with plausibly no worsening over time [6]. The recent availability of anti-amyloid monoclonal antibodies, which have shown efficacy in early symptomatic stages of Alzheimer’s disease, highlights the need to better define early cognitive trajectories [7,8,9]. A refined characterization of prodromal stages may improve our understanding of disease progression and help identify individuals who could benefit from timely intervention as therapeutic options evolve.

In Brazil, around 29% of people aged 50 years and older are estimated to report SCD [10]. Data from several low-, middle- and high-income countries show an average SCD prevalence of almost 24%, with wide variability ranging from 6.1% to 52.7% [11,12]. This heterogeneity may be related to different classification methods or socioeconomic conditions [12]. However, the high prevalence of this condition highlights the need for prevention strategies to counteract its risk factors.

There is ongoing debate in the scientific literature regarding whether modifiable risk factors for dementia are also applicable to SCD [1], and whether some of these factors may play a more prominent role during the initial stages of the natural history of cognitive decline [1]. Indeed, the genetic framework for dementia prevention still has many gaps and modifiable risk factors—including those related to diet, physical activity, cognitive training, and smoking habit—seem to play a role also in genotypes with an increased risk of dementia development [1,13]. In addition, the impact of these risk factors may vary according to the social context, either due to the interactions between multilevel social determinants or differences in exposure intensity [1,14]. Therefore, it is essential to broaden the research on SCD beyond high-income regions. In countries like Brazil, factors such as economic instability, ethno-racial disparities, restricted access to formal education, and adverse working conditions can negatively affect cognitive reserve [15]. Understanding SCD risk factors in low- and middle-income countries (LMICs) helps inform public health policies [14]. Despite being an upper-middle-income country, Brazil shows internal disparities between regions [16]. Furthermore, Brazil is experiencing a rapid population aging process compared to countries with a stable demographic pattern [14]. Therefore, this study aims to investigate the prevalence of SCD and its relationship with well-known risk factors for dementia—including sociodemographic, lifestyle and health-related factors—in a nationally representative sample of Brazilian adults.

## 2. Materials and Methods

### 2.1. Study Design and Study Population

This is a cross-sectional study based on the Brazilian longitudinal study of aging (ELSI-Brazil), a Brazilian cohort study of subjects aged ≥50 years, collecting data in 2015–2016 (wave 1) and 2019–2021 (wave 2). This study is based on data collected in wave 2 on a total sample of 9949 subjects [17]. The ELSI-Brazil is a nationally representative population-based longitudinal research initiative on human aging throughout Brazil, carried out by the Federal University of Minas Gerais and the Oswaldo Cruz Foundation [17]. A multi-stage stratified sampling method ensured representativeness [17]. Sampling included stratified municipalities by size, followed by census and households.

The study was approved by the Research Ethics Committee of the René Rachou Institute/FIOCRUZ Minas (CEP/CONEP) on 29 November 2022, and the process is registered on Plataforma Brasil (CAAE: 34649814.3.0000.5091) [17]. Patient consent was waived due to this study being a secondary analysis of the ELSI-Brazil research database, and informed consent was obtained from participants through database-based declarations.

### 2.2. Data Collection

ELSI-Brazil used face-to-face household and individual questionnaires, administered by trained interviewers. In particular, the individual questionnaire included modules on sociodemographic characteristics, environment and neighborhood conditions, discrimination experiences, life and health history, functionality, employment and retirement, family support, behaviors, general health aspects and diseases, and cognitive assessments. Among the medical conditions investigated, cardiovascular, metabolic, musculoskeletal, and mental health diseases were assessed through self-reported responses.

### 2.3. Study Variables

The study outcome was the presence of SCD (yes or no), defined as self-perceived cognitive decline in the absence of objective cognitive impairment in psychometric tests and without a diagnosis of dementia. Specifically, SCD was determined based on the combination of two questions: response “fair” OR “bad” (excluding those who answered “good”, “very good” and “excellent”) to the question “Currently, how do you classify your memory?” AND response “same” OR “worse” to the question “Comparing your memory to how it was 2 years ago, how do you think that your current memory is?” [18].

To exclude individuals with possible objective cognitive impairment, a verbal fluency test was used as an exclusion criterion. The test was based on the number of animal names recalled by the participant during a timed period. Cut-off points were adjusted for education level, <9 for illiterate individuals, <12 for those with 1–3 or 4–7 years of schooling, and <13 for those with eight or more years of schooling.

Exposures of interest were selected based on known risk factors for dementia as established by Livingston et al. [1], along with sex, age, and ethnicity. Variables were classified into social factors, lifestyle behaviors and health-related factors.

For social factors, we included the following:

- Educational level, categorized according to the International Standard Classification of Education (ISCED) [19] as no education (0), primary (1), lower secondary (2), upper secondary (3), and tertiary/university degree (4–8);
- Per capita income, categorized into low, lower-middle, upper-middle/high, according to the World Bank classification [20];
- Loneliness, categorized into never, almost or always, based on self-reported frequency of feeling lonely.

For lifestyle factors, we included the following:

- Physical activity, classified as sedentary (vigorous activity < 75 min/week or moderate activity < 150 min/week) or active [21];
- Smoking habit, categorized as never, former or current smoker;
- Alcohol consumption, categorized as no/yes for alcohol abuse, is defined as >21 units of alcohol per week [22].

For health-related factors—based on self-reported prior medical diagnosis of chronic diseases, and a subset of them, known as risk factors for cognitive decline—were considered:

- hypertension;
- diabetes;
- hypercholesterolemia;
- hearing loss;
- visual loss;
- obesity.

In particular, hearing loss was identified as self-reported hearing quality, i.e., answering “bad” or “very bad”, or difficulty listening to ambient sounds. Visual loss was defined as difficulty seeing near or far, even with glasses, or a self-reported diagnosis of any eye disease. Obesity was defined as body mass index (BMI) ≥ 30 kg/m^2^.

For each disease, participants were classified into three categories: (i) no diagnosis; (ii) diagnosis reported as the only chronic disease; and (iii) diagnosis reported with at least one additional chronic disease. Furthermore, a variable representing the total number of chronic diseases was created and used as a proxy for multimorbidity.

Finally, depressive symptoms were considered, assessed using the eight-item Centre for Epidemiological Studies Depression Scale (CES-D8). Participants were classified as no (CES-D8 < 4) or “yes” (CES-D8 ≥ 4) [23].

### 2.4. Statistical Analysis

Weighted prevalence of SCD and 95% confidence intervals (CI) were estimated considering the whole study population and strata of sex and age group (i.e., 50–60, 60–79, 80+). Weighted percentages were used to describe the distribution of the variables of interest, overall and according to the study outcome (i.e., SCD). Differences between groups were verified through the chi-squared test for categorical variables or the *t*-test for continuous ones.

To assess and quantify the association between the selected social, lifestyle, and health-related factors and SCD, odds ratios (ORs) and corresponding 95% CI were estimated through adjusted logistic regression models, using the quasi-binomial family to estimate the parameters. Two adjusted models were used: Model 1 adjusted for sex, age, ethnicity, and education level. Model 2 further adjusted for Model 1 plus number of chronic diseases, physical activity, and loneliness.

Stratified analyses were performed by sex, age (<median; ≥median), and educational level (“low” combining together the categories no education and primary education and “middle/high” combining lower secondary or higher education levels). Heterogeneity between strata was evaluated by using the likelihood ratio test comparing models with and without the interaction terms between the exposure and stratification variable.

A sensitivity analysis was conducted using an alternative definition of SCD proposed by Borelli et al. [10], excluding from the analysis individuals reporting moderate to severe depressive symptoms (i.e., CES-D8 ≥ 4). This aimed to verify the consistency of our findings when applying a more restrictive SCD definition that reduces the potential overlap between depressive symptoms and SCD.

In all analyses, a significance level of 5% was admitted minimizing possible type I errors. The data were analyzed using the “survey” package from the R^®^ software, version 2024.4.2.

## 3. Results

**Figure 1:**
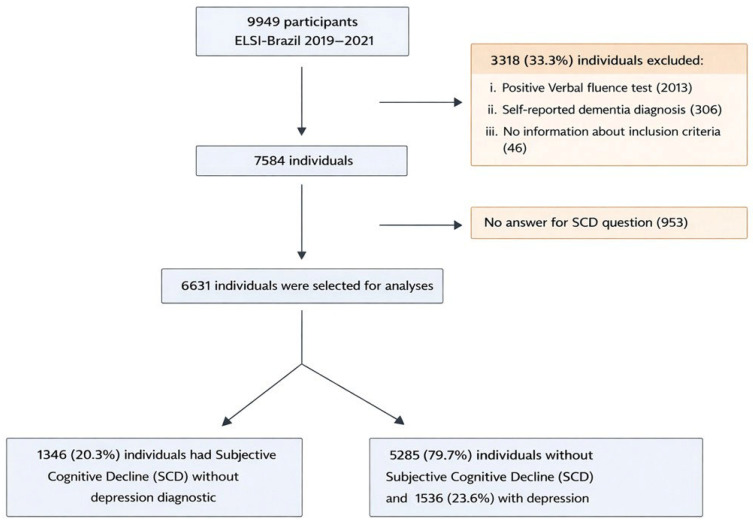
Flow chart of the study participants’ selection.

The study sample selection is reported in Figure 1. Out of the initial sample of 9949 individuals, 2319 were excluded due to the presence of cognitive impairment measured by a positive verbal fluency test and/or self-reported medical diagnosis of dementia, and 46 were excluded due to missing information on the verbal fluency test. A total of 953 were excluded due to missing outcome data. Therefore, the final study sample included 6631 individuals.

Table 1 gives the weighted prevalence and 95% CI of SCD overall, by sex and age group. Overall SCD prevalence was 19.7 (95% CI: 18.6–20.9), and this prevalence increased by age (28.8% at age 80+).

Table 2 gives the distribution of selected characteristics and potential associated factors in the study population, overall and by outcome. Overall, approximately 58% (*n* = 3864) of the whole study population were women, 54.4% (*n* = 3600) were of non-white race, and the average age was 65.1 years (standard deviation, SD: ±9.70 years).

In unadjusted comparisons, individuals with SCD were older, more frequently white, and had lower educational and income levels compared with those without SCD. They also more frequently reported loneliness and a sedentary lifestyle. Regarding health-related factors, multimorbidity and chronic diseases, including hypertension, diabetes, hypercholesterolemia, visual and hearing loss, and depressive symptoms, were more frequent among participants with SCD. The distribution of each chronic disease in the whole study sample is shown in the Supplementary Figure S1.

Table 3 reports the ORs and corresponding 95% CI estimated from Model 1 (i.e., adjusted for sociodemographic characteristics) and Model 2 (i.e., further adjusted for lifestyle and health-related factors). Supplementary Figure S2 summarizes ORs from the Model 2 for variables significantly associated with SCD. In fully adjusted analysis (Model 2), SCD was significantly associated with increasing age (OR = 1.57 for 80+ vs. 50–60 years old), and non-white ethnicity (OR = 1.53). Statistically significant higher odds of SCD were also reported among individuals with lower education (OR = 2.79 for the lowest vs. the highest level) and lower income (OR = 1.58). Loneliness (OR = 1.35) and sedentary lifestyle (OR = 1.33) were also independently associated with SCD. Among health-related factors, significantly higher odds of SCD were observed for multimorbidity (OR = 1.40 for ≥3 chronic diseases), diabetes (OR = 1.25), visual loss (OR = 1.31), and hearing loss (OR = 2.29). Similarly, depressive symptoms were also significantly associated (OR = 1.30).

The results from stratified analyses by sex, age and education level are reported in Supplementary Table S1. However, no significant heterogeneity was found.

Finally, the sensitivity analysis excluding individuals with moderate to severe depressive symptoms showed results consistent with those of the main analysis, except for hypertension and diabetes, which were no longer associated (Supplementary Table S2).

## 4. Discussion

Our study, based on a large nationally representative sample of Brazilians aged 50 and older, estimated a SCD prevalence of 19.7% (95% CI: 18.6–20.9) in 2019–2021. Higher odds of SCD were observed with older age, non-white ethnicity, lower levels of education, lower income, loneliness, and sedentary lifestyle. Associations with higher odds of SCD were also observed with multimorbidity, as well as with specific chronic diseases—including diabetes, visual loss, hearing loss, and depressive symptoms. No significant heterogeneity was found in the stratified analyses across sex, age group, and education variables, and findings remained consistent when applying an alternative outcome classification.

The prevalence of SCD in Brazil, affecting approximately one-fifth of individuals aged 50 or more years, was almost 10% lower than in the first wave [10]. However, this prevalence should be interpreted taking into consideration the exclusions applied in our study population. Indeed, several individuals were excluded due to objective cognitive impairment identified by the verbal fluency test or reported depression diagnosis, i.e., factors that may have been influenced by the COVID-19 pandemic. Notably, similar findings were observed in a Swiss population-based study, where SCD prevalence remained stable before and during the pandemic, despite increased mental health concerns and social isolation [11]. When compared with international estimates, the Brazilian SCD prevalence results are comparable with the average in high-income countries [3,4,5].

While age is biologically expected to increase vulnerability to cognitive decline, our findings indicate that non-white individuals are often positively associated with SCD. This is in line with evidence from the United States showing ethnic disparities in cognitive health outcomes [24]. These results highlight how structural inequities affect early cognitive impairment.

Among the known risk factors for dementia, educational level is considered one of the earliest in the life course [1] and among the most impactful [25]. Early-life education affects cognitive function in old age [26]. In addition to being a strong predictor of dementia, education also influences progression from SCD to MCI [27]. The association between lower education and SCD supports the need for public policies promoting early-life education to enhance cognitive reserve [27]. It is on this concept of cognitive reserve that public policies to promote the broad and prolonged education of young people in LMICs should be based.

Economic [14] and family contexts [28,29] may influence cognitive decline risk. However, this relationship does not appear to be direct between income and cognitive decline. Instead, income may play a role in shaping an individual’s functionality, facilitating or obstructing access to cognitive capacity developers, and modulating the chance of developing SCD [29]. Income was consistently associated with SCD across education and age, suggesting a link to cognitive decline before dementia onset [29].

Social isolation, typical of advancing ages, differs from loneliness [30]. Loneliness reflects perceived inadequacy of social networks [30]. Both social isolation and loneliness are linked to cognitive decline, with loneliness possibly mediated by depression [30]. Loneliness was linked to SCD even without a depression diagnosis or symptoms, likely suggesting a distinct mediation mechanism in SCD [30].

It was estimated that the effect of regular physical activity on SCD incidence in American adults, identifying running, weight lifting and aerobic exercises practiced for 240 min per week as sufficient to mitigate the onset of SCD, providing support for a potential causal relationship between physical activity and cognitive function [31]. Our findings support regular physical activity as a preventive factor for cognitive decline [31,32]. However, evidence suggests that the relationship between sedentary behavior and SCD is mediated by depression [33], a pathway that could not be evaluated in the present study, as individuals with a confirmed diagnosis of depression were excluded from the SCD group.

Clinically, conditions like hypertension, diabetes, and visual loss are related to dementia [1]. However, there is disagreement about the relationship between SCD and various clinical conditions [34]. Multimorbidity is increasingly recognized as a clinical factor associated with SCD [33], especially in LMICs [35]. In this study, apart from hearing loss, no isolated association was found between dementia risk factors and SCD, such as hypertension, diabetes, hypercholesterolemia, obesity, depressive symptoms, and visual loss. On the other hand, multimorbidity with three or more chronic diseases was associated with SCD. Multimorbidity leads to physical issues and poor self-care (e.g., polypharmacy), thus affecting cognition [32]. These aspects highlight the importance of identifying and monitoring multimorbidity among older individuals.

There is strong evidence that both subjective and objective hearing loss increase the incidence of SCD in high-income countries [36]. Hearing loss mirrors brain changes associated with cognition and varies by severity [36]. Based on our findings, hearing loss was associated with SCD, indicating that hearing loss, as a modifiable factor for cognitive decline, requires specific preventive actions regardless of whether it is accompanied by organic or functional impediments [36]. This is crucial in LMICs, where low education and economic barriers limit access to hearing care.

Overall, preventive interventions may include multidomain lifestyle approaches targeting vascular and metabolic risk factors, physical inactivity, diet, smoking, and social isolation, as well as structured risk-factor control programs in midlife and early late life [1,37]. Case-finding strategies could involve proactive cognitive screening in high-risk individuals and early neuropsychological assessment of subjective cognitive complaints, adapting these strategies to the LMICs settings [38].

This study has both strengths and limitations. Among strengths, first, the ELSI-Brazil data were derived from a large, representative sample of Brazilians aged 50+, thus ensuring generalizability. Second, data collection was conducted using standardized and harmonized instruments, aligned with International longitudinal ageing studies, ensuring comparability across countries. Third, the ELSI-Brazil study provided detailed individual-level data on multiple dimensions (i.e., sociodemographic, psychosocial, and clinical factors), enabling adjustment for known dementia risk factors.

In addition to strengths, some limitations should be considered when interpreting the results. First, due to the cross-sectional design, reverse causality cannot be ruled out, especially for more proximal factors in the natural history of cognitive decline, such as physical activity, loneliness, and depressive symptoms, which may represent consequences rather than causes of SCD. In addition, the cross-sectional design limits the ability to distinguish between mediating and direct effects of the investigated factors on SCD. Second, the ELSI-Brazil wave 2 data were collected during the COVID-19 pandemic, possibly affecting cognition and mental health. Third, as in most population-based studies, self-reported data may introduce recall bias or misclassification. Fourth, our exclusion of objective impairment relied on a single cognitive screening measure (animal verbal fluency), which may under-detect other cognitive domains and may therefore lead to residual misclassification (i.e., possible inclusion of early MCI). Fifth, although we were able to adjust for several confounders, data on specific medications potentially affecting cognition were not available. Therefore, residual confounding related to pharmacological exposure cannot be excluded. Lastly, since the SCD definition is based on subjective self-reported information and ELSI-Brazil did not include a validated multi-item psychometric tool specifically for SCD, we adopted a case definition previously used in studies based on the same data source [2,10].

## 5. Conclusions

SCD in Brazil was associated with older age, non-white ethnicity, lower education and lower income, loneliness, sedentary lifestyle, multimorbidity, and sensory impairment, particularly hearing loss. Conversely, hypertension, diabetes, and depressive symptoms were not independently associated with SCD after adjustment. These population-based findings highlight the multidimensional nature of SCD and support the prioritization of vulnerable populations in healthy ageing policies and public health strategies aimed at reducing social and health inequalities. Longitudinal studies are needed to clarify cognitive trajectories, from SCD to MCI and dementia, and causal pathways.

## Figures and Tables

**Table 1:** Weight Prevalence and 95% confidence intervals of SCD overall, by sex and age group (ELSI-Brazil, wave 2, 2019–2021).

	Total N	SCD n	SCD Weighted Prevalence (95% CI)
Overall	6631	1346	19.7 (18.6–20.9)
Males	2767	538	20.2 (18.7–21.7)
Females	3864	808	20.9 (19.6–22.2)
Age 50–60	2019	336	16.5 (14.5–18.4)
Age 60–79	3956	821	20.4 (18.9–21.9)
Age 80+	656	189	28.8 (24.6–33.0)

**Table 2:** Distribution (frequency and weighted percentage) of 6631 Brazilian individuals aged 50 or older for demographic, social, lifestyle and health-related factors, overall and according to the study outcome (ELSI-Brazil, wave 2, 2019–2021).

		Total N (%)	SCD		*p*-Value *
			No N (%)	Yes N (%)	
Overall		6631	5285 (80.3)	1346 (19.7)	
Sex					
	Women	3864 (57.5)	3056 (79.8)	808 (20.2)	0.20
	Men	2767 (42.6)	2229 (80.9)	538 (19.1)	
Age [50–109 years] Mean (SD)		65.1 (±9.7)	65.0 (±9.7)	65.2 (±9.8)	<0.01
Ethnicity					
	Non-White	3600 (54.4)	2771 (76.9)	829 (15.9)	<0.01
	White	2968 (45.6)	2454 (84.1)	514 (23.1)	
Educational Level					<0.01
	No education	676 (9.9)	471 (69.3)	205 (30.7)	
	Primary	3404 (51.2)	2618 (77.7)	786 (22.3)	
	Lower secondary	1292 (20.3)	1065 (82.3)	227 (17.7)	
	Upper secondary	901 (14.3)	802 (90.0)	99 (10.0)	
	Tertiary/University degree	279 (4.3)	253 (88.10)	26 (11.9)	
Income level					<0.01
Low		704 (11.5)	540 (76.7)	164 (23.3)	
Low-Mild		4424 (70.5)	3420 (77.9)	1004 (22.1)	
Upper-Mild/High		1121 (18.0)	971 (87.1)	150 (12.9)	
Loneliness					<0.01
	Never	3990 (64.8)	3260 (82.5)	730 (17.5)	
	Sometimes/Always	2231 (35.2)	1697 (76.4)	537 (23.6)	
Physical Activity					<0.01
	Active	2646 (43.6)	2198 (83.1)	448 (16.9)	
	Sedentary	3617 (56.4)	2762 (77.0)	855 (23.0)	
Alcohol Abuse					0.21
	No	6520 (98.0)	5190 (80.1)	1330 (19.9)	
	Yes	111 (2.0)	95 (86.4)	16 (13.6)	
Smoking Habits					0.23
	No	5182 (68.0)	3636 (68.7)	904 (66.7)	
	Ex/Current	2402 (32.0)	1642 (31.3)	442 (33.3)	
Number of chronic diseases					<0.01
0		849 (13.4)	729 (86.6)	120 (14.0)	
1		1141 (17.7)	948 (83.6)	193 (16.4)	
2		1152 (18.2)	924 (80.8)	228 (19.2)	
≥3		3333 (50.7)	2552 (77.1)	781 (22.9)	
Hypertension					<0.01
	No	3029 (47.2)	2480 (82.7)	549 (17.3)	
	Yes (alone)	426 (6.6)	357 (84.8)	69 (15.2)	
	Yes (with comorbidities)	3020 (46.2)	2316 (76.9)	704 (23.1)	
Diabetes					<0.01
	No	5298 (82.1)	4240 (81)	1049 (19.0)	
	Yes (alone)	65 (1.1)	59 (87.2)	6 (12.85)	
	Yes (with comorbidities)	1121 (16.9)	854 (75.7)	267 (24.3)	
Obesity					0.76
	No	2358 (42.1)	1873 (80.0)	485 (20.0)	
	Yes	3215 (57.9)	2536 (79.7)	679 (20.3)	
Hypercholesterolemia					0.09
	No	4939 (76.5%0	3973 (80.9)	960 (19.1)	
	Yes (alone)	83 (1.3)	64 (79.4)	19 (20.6)	
	Yes (with comorbidities)	1453 (22.3)	1110 (77.7)	343 (22.3)	
Visual Problems					<0.01
	No	3731 (59.3)	3092 (83.2)	639 (16.8)	
	Yes (alone)	82 (1.3)	67 (83.9)	15 (16.4)	
	Yes (with comorbidities)	2653 (39.4)	1985 (75.4)	668 (24.6)	
Hearing loss					<0.01
	No	4709 (73.7)	3980 (84.8)	729 (15.2)	
	Yes (alone)	145 (2.3)	103 (72.5)	42 (27.5)	
	Yes (with comorbidities)	1589 (24.1)	1041 (66.6)	548 (33.4)	
Depressive symptoms ^†^					<0.01
	No	4588 (77.8)	3759 (82.4)	829 (17.6)	
	Yes (alone)	79 (1.5)	61 (77.3)	18 (22.7)	
	Yes (with comorbidities)	1267 (21.3)	923 (74.3)	344 (25.7)	

Caption: SCD: Subjective cognitive decline; SD: standard deviation. * *p*-value is for chi-squared test for categorical variables and *t*-test for continuous variables (i.e., age). ^†^ based on the CES-D8 scale.

**Table 3:** Estimates of associations (OR and 95% CI) between SCD and demographic, social, lifestyle, and health-related factors (ELSI-Brazil, wave 2, 2019–2021).

	OR Model 1 * (95%CI)	OR Model 2 ^†^ (95%CI)
Sex (ref. male)	0.91 (0.78–1.05)	0.99 (0.84–1.16)
Age		
50–59	1	1
60–79	**1.22 (1.02–1.45)**	**1.22 (1.01–1.47)**
80+	**1.72 (1.33–2.23)**	**1.57 (1.19–2.09)**
P for trend	<0.01	0.006
Ethnicity (ref. white)	**1.56 (1.34–1.81)**	**1.53 (1.30–1.79)**
Educational Level		
No education	**3.19 (2.35–4.33)**	**2.79 (2.02–3.85)**
Primary	**2.32 (1.81–2.97)**	**2.14 (1.65–2.77)**
Lower secondary	**1.79 (1.34–2.37)**	**1.67 (1.24–2.25)**
Upper secondary or higher	1	1
P for trend	<0.01	<0.01
Income level		
Low	**1.55 (1.13–2.12)**	**1.58 (1.14–2.21)**
Lower-Middle	**1.44 (1.14–1.83)**	**1.50 (1.17–1.92)**
Upper-Middle/High	1	1
P for trend	<0.01	<0.01
Loneliness		
Never	1	1
Sometimes/Always	**1.41 (1.20–1.65)**	**1.35 (1.15–1.59)**
Physical Activity		
Active	1	1
Sedentary	**1.36 (1.17–1.60)**	**1.33 (1.13–1.57)**
Alcohol Abuse		
No	1	1
Yes	0.57 (0.29–1.14)	0.60 (0.30–1.20)
Smoking Habits		
No	1	1
Ex/Current	1.09 (0.93–1.27)	1.08 (0.92–1.27)
Number of chronic diseases		
0	1	1
1	1.14 (0.84–1.54)	1.11 (0.81–1.52)
2	1.30 (0.97–1.74)	1.18 (0.87–1.61)
≥3	**1.47 (1.13–1.91)**	**1.40 (1.06–1.84)**
P for trend	<0.01	<0.01
Hypertension		
No	1	1
Yes (alone)	0.85 (0.61–1.17)	0.93 (0.65–1.32)
Yes (with comorbidities)	**1.27 (1.08–1.49)**	1.17 (0.99–1.39)
Diabetes		
No	1	1
Yes (alone)	0.62 (0.25–1.54)	0.67 (0.28–1.63)
Yes (with comorbidities)	**1.29 (1.07–1.56)**	**1.25 (1.02–1.53)**
Obesity		
No	1	1
Yes	1.00 (0.85–1.18)	1.00 (0.85–1.18)
Hypercholesterolemia		
No	1	1
Yes (alone)	1.46 (0.75–2.87)	1.67 (0.82–3.40)
Yes (with comorbidities)	1.15 (0.97–1.37)	1.04 (0.87–1.25)
Visual Loss		
No	1	1
Yes (alone)	0.97 (0.49–1.92)	0.87 (0.39–1.92)
Yes (with comorbidities)	**1.414 (1.21–1.64)**	**1.31 (1.11–1.56)**
Hearing Loss		
No	1	1
Yes (alone)	**2.08 (1.31–3.32)**	**2.11 (1.29–3.44)**
Yes (with comorbidities)	**2.47 (2.11–2.90)**	**2.29 (1.93–2.71)**
Depressive symptoms ^‡^		
No	1	1
Yes (alone)	1.56 (0.79–3.09)	1.58 (0.77–3.24)
Yes (with other diseases)	**1.55 (1.29–1.85)**	**1.30 (1.05–1.60)**

* Model 1 was adjusted for the variables sex, age, ethnicity, and education level. ^†^ Model 2 was further adjusted for the number of chronic diseases, physical activity, and loneliness. ^‡^ based on the Centre for Epidemiological Studies Depression Scale (CES-D8) scale. Caption: OR: Odds ratio; CI: confidence interval. Estimates highlighted in bold are statistically significant.

## Data Availability

The data presented in this study are available on request from Johnnatas Mikael Lopes.

## Supplementary Materials

The following supporting information can be downloaded at https://www.mdpi.com/article/10.3390/neurolint18030042/s1, Supplementary Table S1: Estimates of associations (OR and 95% CI from stratified Model 2) between SCD and social, lifestyle and health-related factors, in subgroups of sex, age (below or above median) and education level; Supplementary Table S2: Results from sensitivity analysis (N = 5285): estimates of associations (OR and 95% CI) between the presence of SCD and demographic, social, lifestyle, and health-related factors. ELSI-Brazil, wave 2—2019–2021; Supplementary Figure S1: Percentage distribution of chronic diseases in the sample of participants; Supplementary Figure S2: Summary of the odds ratios (OR) and corresponding 95% confidence intervals (CI) resulting statistically significant from Model 2.

## Abbreviations

The following abbreviations are used in this manuscript:

BMI	Body Mass Index
CES-D	Centre for Epidemiological Studies Depression Scale
CIs	Confidence Intervals
ELSI-Brazil	The Brazilian Longitudinal Study of Aging
ISCED	International Standard Classification of Education
LMICs	Low- And Middle-Income Countries
MCI	Mild Cognitive Impairment
ORs	Odds Ratios
SCD	Subjective Cognitive Decline
