# Supplementary material for: Subjective Cognitive Decline in Brazilian Adults: Prevalence and Associated Social, Lifestyle, and Health-Related Factors: A Nationally Representative Cross-Sectional Analysis from the ELSI-Brazil Cohort
Source: Neurol Int. 2026 Feb 24;18(3):42. doi: 10.3390/neurolint18030042 (PMC13029690; doi:10.3390/neurolint18030042)

Subjective Cognitive Decline in Brazilian Adults: Prevalence and Associated Social, Lifestyle, and Health-Related Factors: a Nationally Representative Cross-Sectional Analysis from the ELSI-Brazil Cohort

Contents

Supplementary Table S1. Estimates of associations (OR and 95% CI from stratified Model 2) between SCD and social, lifestyle and health-related factors, in subgroups of sex, age (below or above median) and education level. 2

Supplementary Table S2. Results from sensitivity analysis (N = 5,285): estimates of associations (OR and 95% CI) between the presence of SCD and demographic, social, lifestyle and health-related factors. ELSI-Brazil, wave 2 – 2019-2021 4

Supplementary Figure S1. Percentages distribution of chronic diseases in the sample of participants. 6

Supplementary Figure S2. Summary of the odds ratios (OR) and corresponding 95% confidence intervals (CI) resulted statistically significant from Model 2. 7

Supplementary Table S1. Estimates of associations (OR and 95% CI from stratified Model 2) between SCD and social, lifestyle and health-related factors, in subgroups of sex, age (below or above median) and education level.

	Women	Men	<Median age	≥Median age	Low Education	Middle/High Education
	OR (95% CI)	OR (95% CI)	OR (95% CI)	OR (95% CI)	OR (95% CI)	OR (95% CI)
Income level						
Low	**1.79 (1.33-2.42)**	**1.39 (1.11-1.75)**	**1.79 (1.33-2.40)**	**1.45 (1.16-1.81)**	**2.17 (1.62-2.91)**	**2.06 (1.53-2.77)**
Low-Mild	**1.63 (1.30- 2.04)**	**1.63 (1.20-2.22)**	**1.48 (1.17-1.85)**	**1.38 (1.02-1.87)**	**1.85 (1.48-2.30)**	**1.81 (1.46-2.24)**
Upper-Mild/High	1	1	1	1	1	1
Loneliness						
Never	1	1	1	1	1	1
Sometimes/Always	**1.37 (1.18-1.57)**	**1.30 (1.12-1.51)**	**1.33 (1.14-1.54)**	**1.32 (1.14-1.54)**	**1.31 (1.13-1.51)**	**1.40 (1.20-1.62)**
Physical Activity						
Active	1	1	1	1	1	1
Sedentary	**1.35 (1.17-1.56)**	**1.37 (1.18-1.60)**	**1.39 (1.20-1.61)**	**1.40 (1.21-1.63)**	**1.46 (1.26-1.69)**	**1.35 (1.16-1.57)**
Alcohol Abuse						
No	1	1	1	1	1	1
Yes	0.62 (0.33-1.17)	**0.48 (0.24-0.95)**	0.56 (0.30-1.05)	**0.49 (0.24-0.97)**	**0.49 (0.25-0.94)**	**0.50 (0.26-0.97)**
Smoking Habit						
Ex/Current	1.08 (0.93-1.25)	1.08 (0.92-1.26)	1.07 (0.92-1.25)	1.09 (0.94-1.27)	1.08 (0.93-1.25)	1.08 (0.93-1.26)
No	1	1	1	1	1	1
Number of chronic diseases	**1.09 (1.03-1.16)**	**1.07 (1.01-1.14)**	**1.08 (1.02-1.15)**	**1.10 (1.04-1.17)**	**1.13 (1.07- 1.20)**	**1.10 (1.03-1.17)**
Hypertension						
No	1	1	1	1	1	1
Yes (alone)	0.95 (0.69-1.32)	0.90 (0.64-1.27)	0.97 (0.70-1.35)	0.92 (0.65-1.29)	0.90 (0.65-1.25)	1.02 (0.73-1.42)
Yes (with comorbidities)	**1.18 (1.01-1.37)**	1.16 (0.99-1.36)	**1.19 (1.02-1.39)**	**1.23 (1.05-1.43)**	1.14 (0.98- 1.32)	**1.24 (1.06-1.45)**
Diabetes						
No	1	1	1	1	1	1
Yes (alone)	0.64 (0.28-1.46)	0.63 (0.26-1.52)	0.60 (0.25-1.43)	0.70 (0.31-1.61)	0.66 (0.28-1.56)	0.61 (0.25-1.50)
Yes (with comorbidities)	**1.29 (1.08-1.54)**	**1.30 (1.07-1.57)**	**1.33 (1.11-1.59)**	**1.29 (1.07-1.54)**	**1.33 (1.11-1.58)**	**1.22 (1.01-1.47)**
Hypercholesterolemia						
No	1	1	1	1	1	1
Yes (alone)	1.79 (0.96-3.34)	1.89 (0.99-3.60)	1.81 (0.99-3.30)	1.69 (0.85- 3.34)	1.62 (0.87-3.03)	1.77 (0.96-3.28)
Yes (with comorbidities)	1.05 (0.89-1.23)	1.02 (0.86-1.21)	1.05 (0.88- 1.24)	1.03 (0.87-1.22)	1.01 (0.86-1.20)	1.02 (0.86-1.21)
Visual Loss						

No	1	1	1	1	1	1
Yes (alone)	0.84 (0.39-1.79)	1.29 (0.67-2.46)	1.12 (0.57-2.19)	0.99 (0.48- 2.04)	0.95 (0.47-1.92)	1.21 (0.62-2.33)
Yes (with comorbidities)	**1.39 (1.19-1.62)**	**1.31 (1.11-1.54)**	**1.41 (1.21- 1.65)**	**1.39 (1.19- 1.62)**	**1.37 (1.17-1.60)**	**1.33 (1.13-1.57)**
Hearing Loss						
No	1	1	1	1	1	1
Yes (alone)	**2.17 (1.38-3.42)**	**1.92 (1.23- 3.00)**	**2.01 (1.27-3.16)**	**2.00 (1.28-3.14)**	**2.05 (1.31-3.20)**	**2.17 (1.39-3.40)**
Yes (with comorbidities)	**2.31 (1.98- 2.69)**	**2.35 (2.00-2.77)**	**2.40 (2.05-2.82)**	**2.36 (2.02-2.76)**	**2.46 (2.11-2.87)**	**2.36 (2.00-2.77)**
Depressive symptoms*						
No	1	1	1	1	1	1
Yes (alone)	1.25 (0.63-2.49)	**1.88 (1.01-3.53)**	1.34 (0.71-2.52)	1.63 (0.83-3.19)	1.44 (0.75-2.76)	1.57 (0.83-2.97)
Yes (with comorbidities)	**1.32 (1.09- 1.59)**	**1.33 (1.09-1.64)**	**1.37 (1.12- 1.66)**	1.21 (1.00- 1.47)	1.40 (1.16-1.70)	**1.29 (1.06-1.58)**
Obesity						
No	1	1		1	1	1
Yes	1.04 (0.89-1.21)	1.04 (0.89-1.22)	1.03 (0.88-1.20)	1.04 (0.89-1.21)	1.07 (0.92-1.24)	1.08 (0.93-1.26)

*based on the Centre for Epidemiological Studies Depression Scale (CES-D 8) scale.

Model 2: Logistic regression model adjusted for sex, age, ethinicity, education level, number of chronic diseases, physical activity, and loneliness.

Caption: OR: Odds ratio; CI: confidence interval; SCD: Subjective cognitive decline.

Supplementary Table S2. Results from sensitivity analysis (N = 5,285): estimates of associations (OR and 95% CI) between the presence of SCD[a] and demographic, social, lifestyle and health-related factors. ELSI-Brazil, wave 2 – 2019-2021.

	OR[b] Model 1 (95%CI)	OR[c] Model 2 (95%CI)
Sex (ref. male)	0.94 (0.78-1.12)	1.01 (0.83-1.22)
Age		
<60	1	1
70-79	**1.39 (1.11-1.73)**	**1.29 (1.03-1.62)**
80+	**1.92 (1.37-2.69)**	**1.66 (1.17-2.34)**
P for trend	*<0.01*	*<0.01*
Ethinicity (ref.white)	**1.52 (1.26-1.83)**	**1.51 (1.25-1.83)**
Educational Level		
No education	**3.00 (2.04-4.40)**	**2.81 (1.90-4.15)**
Primary	**2.27 (1.68-3.08)**	**2.14 (1.56-2.92)**
Low secondary	**1.98 (1.40-2.79)**	**1.93 (1.35-2.76)**
Upper secondary of higher	1	1
P for trend	*<0.01*	*<0.01*
Income level		
Low	1.40 (0.94-2.09)	1.49 (0.99-2.25)
Low-Mild	**1.43 (1.07-1.90)**	**1.50 (1.12-2.01)**
Upper-Mild/High	1	1
P for trend	*0.04*	*0.02*
Loneliness		
Never	1	1
Sometimes/Always	**1.36 (1.11-1.67)**	**1.35 (1.09-1.66)**
Physical Activity		
Active	1	1
Sedentary	**1.49 (1.23-1.81)**	**1.49 (1.22-1.81)**
Alcohol Abuse		
No	1	1
Yes	0.69 (0.32-1.48)	0.65 (0.30-1.41)
Smoke Habit		
No	1	1
Ex/Current	1.06 (0.87-1.28)	1.04 (0.86-1.28)
Chronic diseases		
0	1	1
1	1.06 (0.75-1.49)	1.03 (0.72-1.46)
2	1.28 (0.91-1.80)	1.20 (0.85-1.70)
≥3	**1.48 (1.10-2.01)**	**1.44 (1.06-1.97)**
P for trend	*0.01*	*0.02*
Hypertension		
No	1	1
Yes (alone)	0.81 (0.56-1.19)	0.92 (0.62-1.36)
Yes (with comorbidities)	**1.25 (1.02-1.53)**	1.18 (0.96-1.45)
Diabetes		
No	1	1
Yes (alone)	0.58 (0.19-1.73)	0.60 (0.20-1.75)
Yes (with comorbidities)	**1.32 (1.03-1.68)**	1.27 (0.99-1.62)
Hypercholesterolemia		
No	1	1
Yes (alone)	1.26 (0.56-2.82)	1.38 (0.60-3.16)
Yes (with comorbidities)	1.20 (0.96-1.50)	1.10 (0.88-1.38)
Visual Loss		
No	1	1
Yes (alone)	0.59 (0.19-1.82)	0.62 (0.19-1.99)
Yes (with comorbidities)	**1.34 (1.11-1.62)**	**1.27 (1.03-1.56)**
Hearing Loss		
No	1	1
Yes (alone)	**2.25 (1.32-3.85)**	**2.43 (1.41-4.20)**
Yes (with comorbidities)	**2.52 (2.06-3.08)**	**2.36 (1.92-2.91)**
Obestiy		

No	1	1
Yes	1.02 (0.84-1.25)	1.02 (0.83-1.25)

[a]Individuals with SCD without a diagnosis of depression and a CESD D8 score of less than 4.
[b]Model 1: Logistic regression model adjusted for sex, age, ethnicity, and education level.
[c]Model 2: further adjusted for number of chronic diseases, physical activity, and loneliness.
Caption: OR: Odds ratio; CI: confidence interval; SCD: Subjective cognitive decline.

Supplementary Figure S1. Percentages distribution of chronic diseases in the sample of participants.

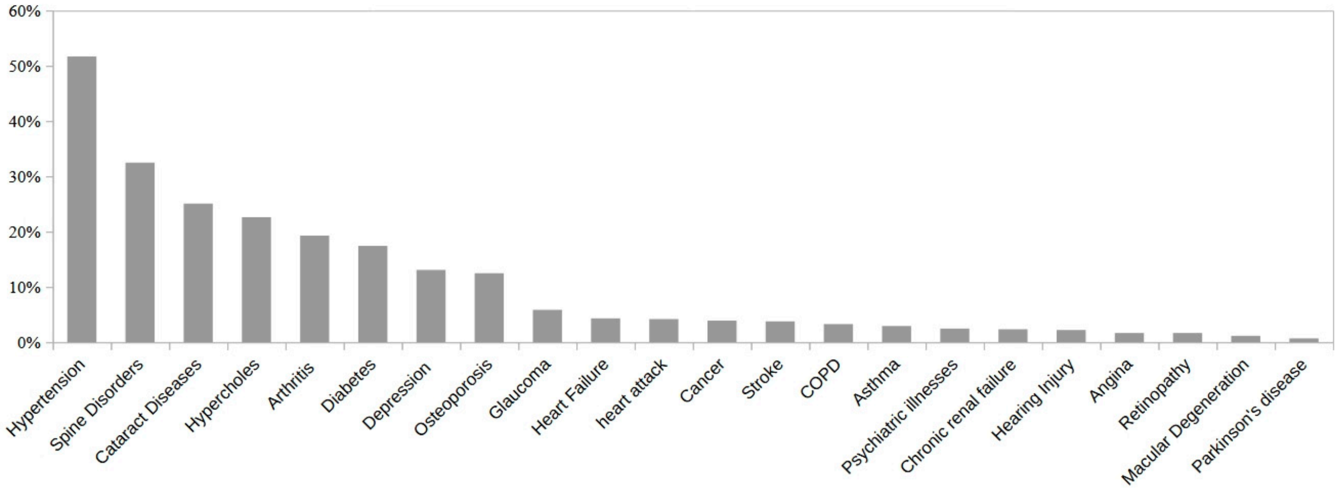

Supplementary Figure S2. Summary of the odds ratios (OR) and corresponding 95% confidence intervals (CI) resulted statistically significant from Model 2.

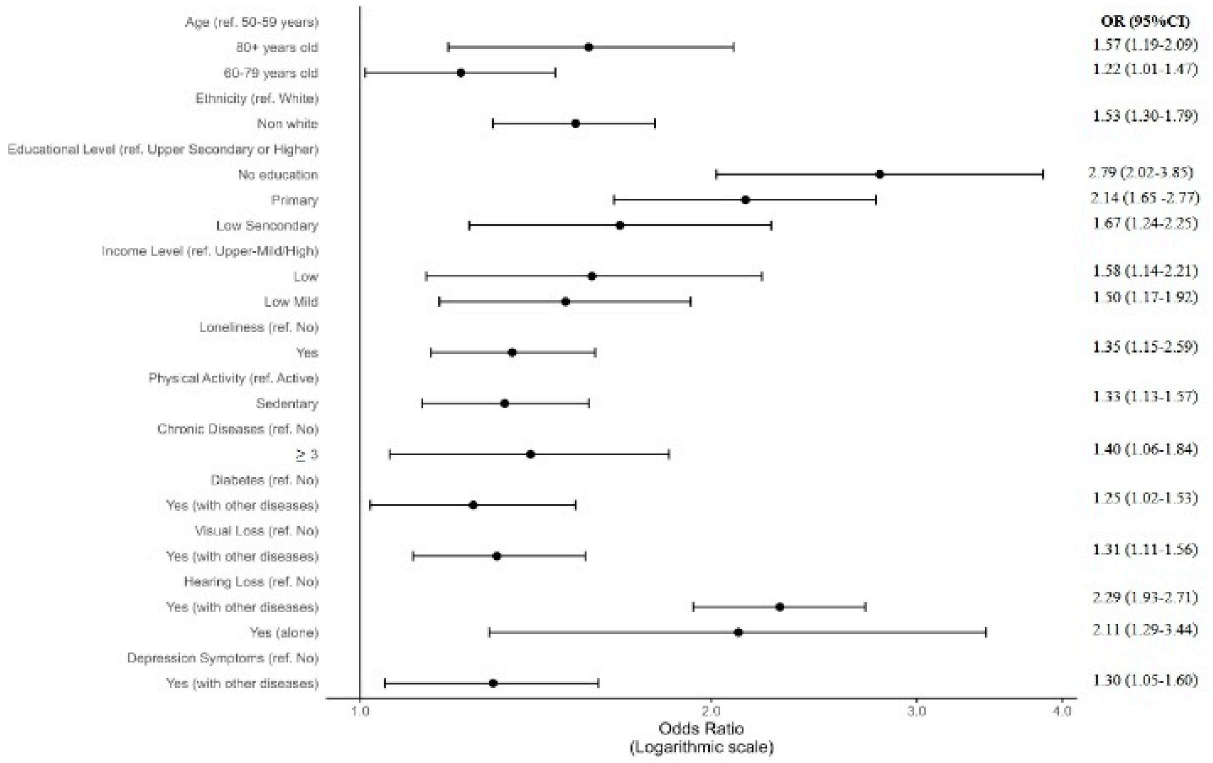

Supplement: Supplementary file 1 [file neurolint-18-00042-s001.zip › neurolint-4137652-supplementary.pdf]
